# POLG1-Related Epilepsy: Review of Diagnostic and Therapeutic Findings

**DOI:** 10.3390/brainsci10110768

**Published:** 2020-10-23

**Authors:** Nicola Specchio, Nicola Pietrafusa, Costanza Calabrese, Marina Trivisano, Chiara Pepi, Luca de Palma, Alessandro Ferretti, Paolo Curatolo, Federico Vigevano

**Affiliations:** 1 Rare and Complex Epilepsy Unit, Department of Neuroscience, Bambino Gesù Children’s Hospital, IRCCS, Full Member of European Reference Network EpiCARE, 00165 Rome, Italy; nicola1.pietrafusa@opbg.net (N.P.); costanza.calabrese@opbg.net (C.C.); marina.trivisano@opbg.net (M.T.); chiara.pepi@opbg.net (C.P.); luca.depalma@opbg.net (L.d.P.); alessandro.ferretti@opbg.net (A.F.); 2 Child Neurology and Psychiatry Unit, Systems Medicine Department, Tor Vergata University, 00133 Rome, Italy; curatolo@uniroma2.it; 3 Department of Neuroscience, Bambino Gesù Children’s Hospital, IRCCS, Full Member of European Reference Network EpiCARE, 00165 Rome, Italy; federico.vigevano@opbg.net

**Keywords:** mitochondrial diseases, DNA polymerase gamma, *POLG1*, epilepsy, genetic findings, neurophysiologic findings

## Abstract

Background: The clinical spectrum associated with *POLG1* gene mutations ranges from non-syndromic epilepsy or mild isolated neurological signs to neurodegenerative disorders. Our aim was to review diagnostic findings, therapeutic approaches and outcomes of reported cases of epilepsy related to *POLG1* mutation. Methods: The articles for review were identified through a systematic research on PubMed and EMBASE databases from January 2003 to April 2020, searching for the terms “Epilepsy AND *POLG* OR polymerase gamma,” OR “*POLG1*”. Results: Forty-eight articles were selected for review, which included 195 patients. Two main peaks of age at epilepsy onset were found: at ages 1 and 13 years. The most frequent seizure type was myoclonic. The occurrence of Status Epilepticus was reported in 46.4% of cases. Epileptiform and slow abnormalities were most frequently seen over occipital regions. Brain Magnetic Resonance Imaging (MRI) revealed increased T2 signal intensities in thalamic regions. Genetic analysis revealed a prevalence of A467T, W748S and G848S (74.2% of patients) mutations. Survival at 5 years was estimated at very low levels (30.2% of patients). Conclusion: In this review, we included cases with both pediatric and adult epilepsy onset. The analysis of data regarding prognosis showed that survival is related to age at onset of epilepsy.

## 1. Introduction

*POLG1* is an enzyme that ensures accuracy in replication and repair of mitochondrial DNA [1]. Human *POLG1* gene (mitochondrial polymerase gamma) was localized by Fluorescence In Situ Hybridization (FISH) to chromosome band 15q24-->q26 in 1997 [2], and several mutations have been reported [3]. Inherited mitochondrial disorders are most commonly caused by POLG mutations (accounting for 10% of adult mitochondrial disease cases), with as many as 2% of the population carrying these mutations [4]. Some POLG mutations cause mitochondrial DNA (mtDNA) depletion syndromes, either in early childhood or later-onset [5].

POLG mutations account for at least six major syndromes and comprise a continuum of overlapping phenotypes with onset from infancy to late adulthood. Alpers–Huttenlocher syndrome (AHS) is characterized by childhood-onset progressive and severe encephalopathy with intractable epilepsy and hepatic failure. Individuals with childhood myocerebrohepatopathy spectrum (MCHS) present with developmental delay, lactic acidosis, myopathy and hepatic impairment. Myoclonic epilepsy myopathy sensory ataxia (MEMSA) encompasses a spectrum of disorders with epilepsy, myopathy and ataxia (typically without ophthalmoplegia) including disorders previously described as spinocerebellar ataxia with epilepsy (SCAE); long-term survivors of MEMSA can additionally develop progressive external ophthalmopegia (PEO). The ataxia neuropathy spectrum (ANS) includes mitochondrial recessive ataxia syndrome (MIRAS) and sensory ataxia neuropathy dysarthria and ophthalmoplegia (SANDO). Autosomal recessive PEO (arPEO) is characterized by progressive weakness of the extraocular muscles, resulting in ptosis and ophthalmoparesis without associated systemic involvement. Autosomal dominant PEO (adPEO, previously listed disorders are all autosomal recessive) typically includes generalized myopathy and variable degrees of sensorineural hearing loss, axonal neuropathy, ataxia, depression, parkinsonism, hypogonadism and cataracts [6,7,8,9,10].

Deficient Pol γ activity in the skeletal muscle and liver of patients with AHS was first reported by Naviaux et al. in 1999 [11], but POLG mutations were not described until 2001. In a multinational cohort, 70% of children with POLG mutations presented with AHS. Disease onset is typically around the end of the first year of life; however, clinical presentation can occur at any time in childhood, and adult onset has even been reported. Onset of seizures is frequently explosive, and most patients present with refractory convulsive status epilepticus (SE). The disease course is characterized by recurrent episodes of SE and epilepsia partialis continua (EPC), and the most frequent cause of death in childhood is liver failure”. Development is often normal, but some individuals who present with AHS have a history of prior hypotonia and mild developmental delay.

The aim of the present article is to review clinical, genetic, neurophysiological, neuroimaging, neuropsychological and histopathology findings; therapeutic approaches; and outcomes of all the reported cases of epilepsy associated with *POLG1* mutations. Our intent was to determine a paradigm for patients’ profiles, which may help in early diagnosis, genetic counseling and treatment for pediatric neurologists.

## 2. Materials and Methods

Two authors (N.P. and N.S.) performed a search of PubMed and EMBASE databases from January 2003 to April 2020, looking for the terms “Epilepsy AND *POLG OR polymerase gamma*,” *OR* “*POLG1*”. References were also identified manually from relevant articles and searching through the authors’ files. Only papers published in English were reviewed. As inclusion criteria, we selected articles in which seizure semiology and/or electroencephalogram (EEG) findings were comprehensively reported. Papers were excluded if age at epilepsy onset and genetic findings were not available. Only patients with pathogenic recessive POLG variants in either a homozygous or compound heterozygous state were considered.

We collected data on sex, ethnicity, familial history, genetic findings, age at seizure onset, seizure semiology, serum lactate, brain MRI findings, muscle biopsy, treatment, neurological and cognitive assessment at onset and during follow-up/outcome.

Data were stratified into four groups according to age at onset of epilepsy (Group 1: age at onset ≤3 years; Group 2: age at onset >3–≤6 years; Group 3: age at onset >6–≤16 years; Group 4: age at onset >16 years) to investigate whether age at onset was associated with disease course and outcome in *POLG1* patients. In order to evaluate the effect of puberty on epilepsy severity, EEG and neuroimaging findings, Group 3 (age >6–<16 years) was stratified into two groups according to age >6–<12 years and >12–<16 years.

### Statistical Analysis

Continuous data were summarized using descriptive statistics including means, standard deviations, medians and ranges. Categorical variables were summarized with frequencies and percentages.

Probability test were planned a priori. For categorical results, a chi-square test or the Fisher’s exact test were performed, as appropriate. Wilcoxon signed-rank test was used for continuous variables. Survival was analyzed with a product-limit method (Kaplan–Meier curves) and comparisons with the log-rank test. Statistical Package for Social Sciences (IBM SPSS Statistics for Window, version 21–IBM Corp., Armonk, NY, USA) was used; a *p*-value < 0.05 was considered statistically significant.

## 3. Results

The initial search from combined databases returned 228 articles (PubMed = 118 and Embase = 110). A total of 48, including 195 cases, met the inclusion criteria and were used for further evaluation [12,13,14,15,16,17,18,19,20,21,22,23,24,25,26,27,28,29,30,31,32,33,34,35,36,37,38,39,40,41,42,43,44,45,46,47,48,49,50,51,52,53,54,55,56,57,58,59,60].

### 3.1. Demography

One hundred and ninety-five patients who manifested epilepsy related to mutations in *POLG1* gene have been reported since the first description in 2001 [32]. Women were more frequently affected (58.9%), and while female patients had a higher median age at disease onset than male patients, distribution across the sexes was even for onset during the first 6 years of life (Table 1). Age at epilepsy onset ranged from 0 to 66 years (Table 1). Two main peaks of age at onset were presented: at 1 year in 39.3% of patients and at 13 years in 34.0% of patients. A later onset was less frequently reported.

### 3.2. Clinical Features

Different types of seizures were experienced during the disease (Table 2). Overall, the most frequent seizure type was myoclonic, which was found in 95 of 183 cases (51.9%), followed by focal to bilateral tonic clonic seizures (81/183 cases, 44.3%) and focal motor onset seizures (62/183 cases, 33.9%). The occurrence of SE was reported in 85 of 183 cases (46.4%). Recurrent SE was reported in 16 cases (9.7%).

Considering groups, myoclonic seizures and EPC were the most frequent seizure types reported in Group 1, both in 41.3% of cases. Focal motor onset seizures, focal to bilateral tonic clonic seizures and SE were more frequent in Groups 3 and 4 (51.8%, 76.5% and 70.4% of patients, respectively). A minority of patients experienced other types of seizures, such as atonic, non-convulsive SE and focal onset seizures with visual symptoms; however, we cannot exclude that younger patients experience visual symptoms that might be poorly perceived by children.

EPC and SE occurred more often in patients aged >12 years. Interestingly, females were more exposed to EPC and SE if compared with male >12 years (respectively, 72.2% and 61.6% of female versus 54.8% and 48.0% of male patients).

Almost all patients had psychomotor regression and different additional neurological symptoms (96.4%): ataxia 53.6% (60/112 cases reported cases) most commonly, followed by neuropathy 29.4%, hypotonia 21.4%, visual disturbance 18.7%, nystagmus 17% and focal deficits 9%. A normal neurological status before seizure onset was reported in 42.4% of subjects; therefore, epilepsy was the first clinical manifestation in these patients.

### 3.3. EEG Findings

A peculiar EEG feature in patients with Alpers syndrome, and in other forms of *POLG1*-related epilepsy is represented by a Rhythmic High-Amplitude Delta with Superimposed (poly) Spikes (RHADS). This pattern was more evident in patients belonging to Group 1 (15/46 patients, 32.6%). In this group, a reduction in background activity was also frequent (27/46 patients, 58.8%) (Table 2). RHADS were more frequent in pre-puberty without differences in gender. Regarding localization, both epileptiform and slow abnormalities were most frequently seen over occipital regions (19.5% and 14.3%, respectively). Epileptiform abnormalities were more common than slow abnormalities: 44% vs. 22% of patients. EEG findings included burst suppression pattern in patients with early onset disease.

### 3.4. Neuroimaging Findings

Neuroimaging data are summarized in Table 3. Brain MRIs revealed increased T2 signal intensities in thalamic regions (32/109 pts, 29.3%). Brain atrophy was more frequent in Group 1 (20/57, 30.1%) (see Table 3). Focal abnormalities were characterized by T2-hyperintensities in cortical regions, mainly in the occipital areas. White matter, basal ganglia and cerebellum were also involved. Bilateral thalamus and bilateral occipital hyperintensities were more evident in patients with age at onset later than 6 years. Between pre- and post-puberty, we did not find any difference considering neuroimaging findings. Conversely, brain atrophy was significantly more evident in patients younger than 6 years at onset. Magnetic Resonance Spectroscopy (MRS) was documented in 19 patients (11.6%), and 9 of those (47.4%) had a lactate peak. Evaluation of MRI findings was not available in the reported case series; however, we argue that atrophy can be seen between 3 and 6 months from the onset in childhood population.

### 3.5. Genetic Findings

Genetic analysis revealed a large number of mutations, which divide into two main groups: compound heterozygous and homozygous mutations. There was a high prevalence of A467T, W748S and G848S mutations. A complete panel of the other genetic data is available in Supplementary Materials Table S1.

Occurrence of EPC was associated with compound heterozygosity in which one of the mutated alleles was A467T or W748S (*p* < 0.001 and *p* = 0.003, respectively), though statistical bias due to these being the most common mutations cannot be discounted and should be considered as a limitation of the currently available data from literature.

### 3.6. Laboratory Findings

Data about muscle biopsy were reported in 49.6% of cases (72/195), and 63.1% of these revealed abnormal results. Considering age at onset of disease, 61.0% of patients with early onset (0–6 years) had abnormal biopsy findings compared with 51.6% of patients with late-onset disease (>6 years), and 31.5% of patients under the age of 3 years had an abnormal biopsy (Supplementary Materials Table S2). The most frequent finding was cytochrome oxidase-negative fibers on muscle biopsy (42.5%), followed by mild nonspecific changes of fibers (18.5%), abnormal mitochondria (15%), ragged red fibers, fiber size variation and steatosis (7.2%). Levels of lactate were elevated in serum/CSF/urine in 62 (70.0%) of the 89 patients tested (Supplementary Materials Table S2). Lactate was searched in 66 cases (74%) in blood, in 45 cases (50.5%) in CSF and in 9 cases (10%) in urine.

### 3.7. Treatment and Outcome

A large amount of antiseizure medication (ASM) was used in the majority of the patients, though this was ineffective in almost all cases. Valproate was administered to 40 of 56 reported patients (71.4%). In the majority of reported patients (68.7%), the use of valproate was associated with liver problems/dysfunction, ranging from abnormal liver enzymes and liver enlargement (76.2%) to severe acute hepatic failure (39.7%). Hepatic failure might lead to liver transplantation or provoke fatal events within weeks/months after valproate treatment and might persist also after drug withdrawal in some patients [12,13,14,15,16,17,18,21,33,34,35,36,37,38,39,40,41,43,61,62]. Reported treatments also included immune therapy (IVIG and steroids) and ketogenic diet (Supplementary Materials Table S3).

**Figure 1:**
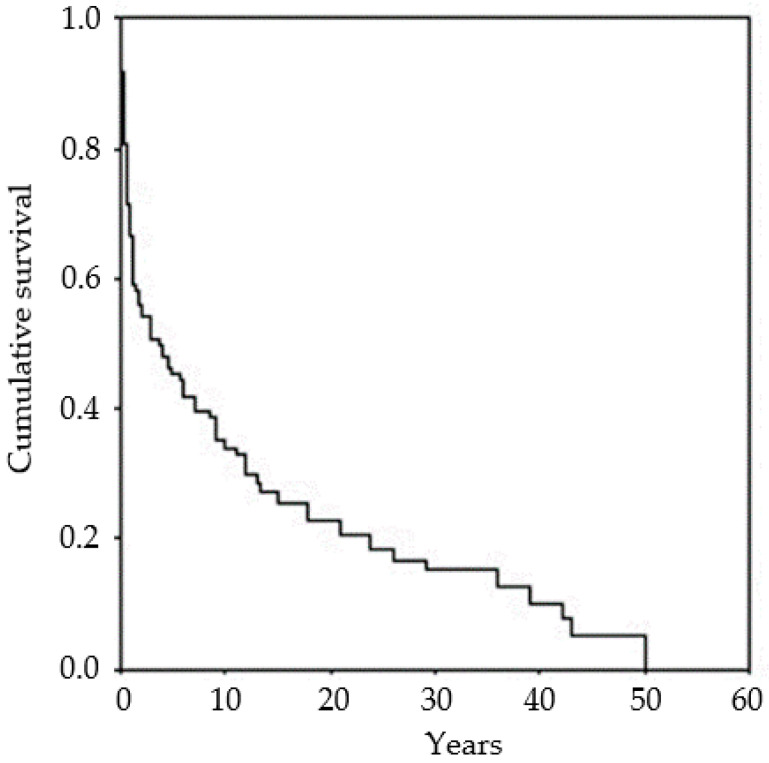
Kaplan–Meier survival curve. Overall survival was 11.5 years (mean, 95% Confidence Limits 8.5–14.5).

Survival at 5 years was estimated at very low levels, with a better outcome in late onset cases. Overall survival was 11.5 years (mean, 95% Confidence Limits 8.5–14.5). Occurrence of EPC was associated with the worst outcome (*p* < 0.01). Survival was expected to be worse in patients belonging to Group 1 (mean 1.9 years), and to be better in patients of Groups 2 (22.2 years), 3 (17.2 years) and 4 (15.7 years) (*p* < 0.001) (Supplementary Materials Table S4). Figure 1 shows the overall survival of the whole group of patients.

## 4. Discussion

The review of published cases has allowed us to identify and summarize the epileptic phenotype of *POLG*-related epilepsy, particularly focusing on the age at onset, seizure type and outcome. This review included 195 cases affected by epilepsy due to *POLG-1* mutations. We considered only cases in which information about epilepsy and EEG were clearly reported to produce more detailed observations and analyses. We included cases with both pediatric and adult epilepsy onset. When possible, a statistical analysis was applied to reduce the bias related to a subjective evaluation.

*POLG1*-related epilepsy is severe and, in the majority of patients, drug-resistant, with poor outcomes in many cases, including fatalities. The condition is probably underestimated because it is rare, insufficiently documented with video-polygraphic recordings and misdiagnosed. Table 4 summarizes the most important clinical findings in this condition. Onset of epilepsy occurs at all ages, though the disease starts during the first years of life and in adolescence in the majority of patients.

Our review shows the typical presentation is with epileptic seizures, mainly of myoclonic type, with occurrences of SE and EPC also being frequently reported. Additionally, almost all patients present with psychomotor regression and different additional neurological symptoms. The stratification of data in patients >6–<12 years and >12–<16 years interestingly revealed that puberty might have a different clinical course in terms of epilepsy and EEG abnormalities, where older patients were more prone to EPC and SE with a female preponderance and younger patients presented more frequently RHADS at EEG.

Serum lactate and muscle biopsy might suggest potential contributions to diagnosis, as these were reported to be altered in 69.6% and 63.1% of the tested cases, respectively. Normal muscle biopsy findings would not exclude the diagnosis of POLG-related mitochondrial disease, and direct sequencing of the POLG gene should be the gold standard when the clinical feature suggests POLG disease [7].

A variety of epileptic seizures have been reported at the onset and during the disease: myoclonic seizures, focal motor, infantile spasms, EPC and intractable and recurrent SE. We found that the recurrence of specific seizures types seems to be age dependent. Myoclonic seizures and EPC are more frequent during the first years of life and SE and generalized tonic-clonic seizures in elder patients. Epileptiform discharges over the occipital region on EEG are a frequent and early finding in *POLG1*-mutated patient, although EEG findings are widely variable. The peculiar EEG pattern (RHADS) seems to be age related, being more frequent in patients less than 3 years, although data are frequently unreported in the literature. The RHADS EEG pattern, together with the occurrence of myoclonic seizures and EPC, might help in reaching diagnosis and in predicting prognosis in the first years of life.

Brain MRI might be helpful for addressing diagnosis, as it reveals brain atrophy, mostly in the youngest patient groups together with cortical hyperintensities in the posterior regions. An involvement of subcortical structures has also been reported. Our results are consistent with previous observations of a predisposition for occipital lobe involvement in POLG-related epilepsy, in terms of clinical, neurophysiologic and radiologic findings; however, the precise explanation of such preferential involvement remains unknow [63].

*POLG1*-related epilepsy is a syndrome with a wide phenotypic spectrum depending mainly on the genetic background. In the context of different phenotypes and severity of the disease, it is important to identify prognostic predictors. From our review, we underline a possible correlation between genetic findings and clinical expression; thus, we found that a compound heterozygosity is more frequently associated with EPC and SE occurrence.

Seizure management is extremely challenging in patients who harbor POLG variants, and often futile in those presenting with recurrent SE and progressive encephalopathy. Epilepsy is usually characterized by myoclonic seizures and EPC, and a large number of ASMs in almost all cases were ineffective. It remains unclear whether earlier recognition of seizure and aggressive treatment with combined ASDs as well as general anesthesia could alter the clinical outcome in all patients with POLG variants, as a significant number of patients (especially pediatric cases with Alpers disease) eventually succumbed to hepatic failure.

Immune-therapy and ketogenic diet have also been attempted without any evidence of efficacy. The use of i.v. magnesium is an interesting tool because it reduced seizures during a refractory SE in some reported cases. Efficacious treatments reported in the case reports are anecdotal, and such evidence is limited by a small number of patients.

Valproate is an absolute contraindication in patients with POLG-related epilepsy. Valproate can precipitate liver failure in Alpers disease, and this was documented in POLG-mutated adolescent and early-adulthood patients treated with valproate for status epilepticus [41]. Valproate is a histone deacetylase inhibitor but is also known to inhibit fatty acid β-oxidation, which primarily occurs in the liver. Valproate further compromises mitochondrial function in POLG-related disease without directly inhibiting POLG or acting on the DNA replication pathway [15].

The data regarding prognosis showed that survival is related to age at epilepsy onset. The worst outcome was seen in patients whose epilepsy appeared in the first 3 years of life (median 0.7 years); patients whose epilepsy appeared >16 years (median 18.0 years) usually had a better outcome. Considering the hypothesis that the outcome might be influenced by seizure type, the only significant association we found was the occurrence of a worse outcome in the presence of EPC during the course of epilepsy. Moreover, we found no association of genetic findings with the presence of RHADS at EEG.

The major limitation of our findings is the retrospective nature of the study design and data extrapolation from a number of case studies with a different emphasis on clinical or molecular findings. Moreover, the selection of articles where seizure semiology and/or EEG findings were comprehensively reported could have biased the study sample. Selecting articles with specific elements of interest might have resulted in an overestimation of some data reported in this review. In some of the articles considered for this review, some data were missing, and tissues examined were not patient matched in all cases. Moreover, our stratification into four groups yielded small numbers.

We identified some genetic and clinical prognostic factors from this review of published literature. Questions remain unsolved regarding the link between genetic findings and prognosis, not only related to survival, but also to hepatic failure in the presence of valproate, which should be avoided in carriers of POLG genetic variants. Moreover, the reason why an early onset is associated with a worse prognosis remains unclear.

## Figures and Tables

**Table 1:** Demographic and clinical features by age at onset.

Demographic and Clinical Features by Age at Onset					
**Age at Onset (y)**	**N.**	**%**	**Median (Mean ± SD)**		
0–≤3	85	43.6	1 (1.4 ± 0.6)		
>3–≤6	10	5.1	5 (4.8 ± 0.9)		
6–≤16	57	29.2	13 (11.2 ± 2.5)		
>16	43	22.4	19 (24.5 ± 8.5)		
Total	195	100	9 (9.9 ± 8.9)		
**Sex by Age at Onset**	**Total**	**Age at Onset**			
		0–≤3	>3–≤6	>6–≤16	>16
Male	64 (41.0%)	32 (46.4%)	3 (60%)	18 (37.5%)	11 (32.3%)
Female	92 (58.9%)	37 (53.6%)	2 (40%)	30 (62.5%)	23 (67.6%)
Total (Data av *)	156/195	69 (100%)	5 (100%)	48 (100%)	34 (100%)

* 39 missing (20.0%); N., numbers; y, years; SD, standard deviation; av, available.

**Table 2:** Seizure and EEG by age at onset.

Seizure and EEG by Age at Onset					
**Seizure Semiology ^**	**Total**	**Age at Onset**			
		**0–≤3**	**>3–≤6**	**>6–≤16**	**>16**
Data available	183/195 *	(92 pts)	(10 pts)	(46 pts)	(35 pts)
Myoclonic	95	38	9	30	18
SE	85	26	2	31	26
Focal to bilateral	81	14	5	33	29
Focal motor	62	16	4	32	10
EPC	55	38	5	11	1
Visual symptoms	21	1	2	8	10
Focal with impaired awareness	18	12	2	4	0
Clonic/hemiclonic	3	3	0	0	0
NCSE	2	0	0	1	1
Atonic	3	1	0	1	1
Multiple foci	4	2	0	2	0
**EEG Findings ^**	**Total**	**Age at Onset**			
		**0–≤3**	**>3–≤6**	**>6–≤16**	**>16**
Data available	77/195 **	(46 pts)	(4 pts)	(15 pts)	(12 pts)
Slowing down BA	37	27	1	2	7
RHADS	20	15	2	3	0
Occipital epileptiform abnormalities	15	5	0	5	5
Occipital Slow Waves	11	9	2	0	0
Non localizing abnormalities	7	5	1	1	0
Multifocal epileptiform abnormalities	6	4	1	1	0
Cen or Par epileptiform abnormalities	1	6	0	3	2
Epileptiform abnormalities bilateral	5	1	0	4	0
Diffuse epileptiform abnormalities	5	3	0	2	0
Front/Temp Slow Waves	4	2	0	2	0
Burst-Suppression	4	4	0	0	0
PLEDs	1	0	0	1	0
Normal	1	0	0	0	1

^ Not mutually exclusive, * 12 missing (6.1%), ** 118 missing (60.5%), pts, patients, SE, status epilepticus; EPC, epilepsia partialis continua; NCSE, non-convulsive status epilepticus; BA, background activity; RHADS, rhythmic high-amplitude delta with superimposed spikes; cen, central; par, parietal; front, frontal; temp, temporal; PLEDs, periodic lateralized epileptiform discharges.

**Table 3:** Neuroimaging findings by age at onset.

Neuroimaging Findings by Age at Onset					
MR Findings ^	Total	Age at Onset			
		0–≤3	>3–≤6	>6–≤16	>16
Data available *	109/195	(57 pts)	(3 pts)	(27 pts)	(22 pts)
Thalamus bilateral	32	11	0	12	9
Atrophy	29	20	1	4	4
Cortical bilateral occipital	28	10	1	9	8
Cerebellum	16	4	0	6	5
White matter	17	13	0	0	4
Thalamus unilateral	12	3	1	7	1
Cortical unilateral occipital	11	5	0	5	1
Cortical unilateral P-T	9	6	1	1	1
Normal	12	8	0	2	2
Cortical bilateral P-T	8	5	0	1	2
Cortical bilateral frontal	8	5	0	2	1
Basal ganglia	6	3	0	2	1
Cerebellum white matter	4	0	0	2	2
Cortical unilateral frontal	2	1	0	0	1
Hippocampal sclerosis	2	2	0	0	0

* 86 missing (44.1%); ^ Not mutually exclusive; P-T, parietal and temporal.

**Table 4:** Main clinical features in POLG-related epilepsy per age.

Main Clinical Features in POLG-Related Epilepsy per Age								
	Age at Onset							
	0–≤3	%	>3–≤6	%	>6–≤16	%	>16	%
Type of seizures	Myoclonic EPC SE	41.3 41.3 28.2	Myoclonic Focal to bil.	90.0 50.0	Focal to bil. Myoclonic Focal motor	71.7 65.2 69.5	Focal to bil. SE Myoclonic	82.8 74.2 51.4
Neurophysiology	Slowing down BA RHADS	58.7 32.6	RHADS	50.0	Occ. Epil. Abn.	33.3	Occ. Epil. Abn.	41.6
Neuroimaging abn.	White matter Thalamus bil Cortical bil. Occ.	22.3 19.3 17.5	Cortical bil. Occ.	33.3	Thalamus bil Cortical bil. Occ.	44.4 33.3	Thalamus bil Cortical bil. Occ.	40.9 36.3

EPC, Epilepsia Partialis Continua; SE, Status Epilepticus; bil., bilateral; BA, Background Activity; RHADS, Rhythmic High-Amplitude Delta with Superimposed (poly) Spikes; Epil., Epileptiform; Abn., Abnormalities; Occ, Occipital.

## Supplementary Materials

The following are available online at https://www.mdpi.com/2076-3425/10/11/768/s1, Table S1: Genetic findings by age at onset, Table S2: Laboratory findings by age at onset, Table S3: Treatment by age at onset, Table S4: Survival by age at onset.
