# Supplementary material for: POLG1-Related Epilepsy: Review of Diagnostic and Therapeutic Findings
Source: Brain Sci. 2020 Oct 23;10(11):768. doi: 10.3390/brainsci10110768 (PMC7690674; doi:10.3390/brainsci10110768)
Supplement: Supplementary file 1 [file brainsci-10-00768-s001.pdf]

Genetic findings by age at onset				
**Genetic findings**	**Age at onset**			
	0-≤ 3 (47 pts)	*>3-≤ 6 (4 pts)*	*>6-≤16 (15 pts)*	*>16 (10 pts)*
A467T/ Q68X	1	0	0	0
A467T/A467T	2	4	33	19
W748S/novel spl.mut	1	0	0	0
W748S/W748S	3	1	4	7
A467T/L966R	3	0	0	0
A467T/G848S	10	0	0	0
A467T/R852C	2	0	0	0
W748S/G848S	1	1	1	0
A467T/IVS14+1G>A	1	0	0	0
A467T/S305R	1	0	0	0
A467T/E358(A)Del-364X	2	0	0	0
L304R / E1145fsX1173	1	0	0	0
A467T / R574W	2	0	0	0
A467T / G303R	3	0	0	0
W748S / E1143G / R232H	1	0	0	0
W748S / E1143G / M1163R	1	0	0	0
A467T/W1020X	2	0	0	0
W748S-E1143G/G848S	2	0	0	0
A467T /C1188R	1	0	0	0
R1096C/ R1096C	1	0	0	0
G517V (Heteroz)	1	0	1	0
T251I / P587L	1	0	1	0
L304R / L392V/ pR1081dup	1	0	0	0
R227W / A467T	2	0	0	0
A957P / A467T	1	0	0	0
W235X / A467T	1	0	0	0
A467T/T914P	2	1	0	0
W748S/G1211RfsX6	2	0	0	0
L244P / W748S	2	0	0	0
A467T /A957P	1	0	0	0
A467T / E1143G / Q879H / T885S	1	0	0	0
W748S+E1143G / R807C	1	0	0	0
A467T / T914P	2	0	0	0
R722H / V1044A	1	0	0	0
W748S+E1143G / T914P	1	0	0	0
R1096C /Q1236H	1	0	0	0
A467T/C418R	0	1	0	0
A467T / Y282D	0	1	0	0
W748S+Q497H+E1143G / A467T	0	1	0	0
R597W/ R597W	0	0	1	0
L83P/ G888S	0	0	1	0
A467T / W748S	0	0	4	2
W748S	0	0	3	2
R852C / G11D R627P	0	0	1	0
A467T / IVS21+ 2T>C	0	0	1	0
R722H / W748S	0	0	1	0
G517V Heterozygous	0	0	1	1
W748S /T914P	0	0	1	0
W748S / E1143G / A467T	0	0	0	1
A467T	3	0	0	3

A2551G / C3139T	0	0	0	1
R627P Q1236H /L965X, Q1143G	0	0	0	1
W748S/Q1143G	0	0	0	1
G848S / P587L	1	0	0	0
A467T / R417T	1	0	0	0
W748S/E1143G/G1205E	0	0	1	0
A467T / G303R	3	0	0	0
W748S; E1143G + W748S/E1143G	1	0	0	0
A467T/L966R	1	0	0	0

Supplemental Table 1. Genetic findings by age at onset

Laboratory findings by age at onset				
**Muscle biopsy**	**Age at onset**			
	0-≤3	>3-≤6	>6-≤16	>16
Normal	14	2	8	7
Abnormal	24	1	7	9
Total (Data av.*)	38	3	15	16
**Lactate****	**Age at onset**			
	0-≤3	>3-≤6	>6-≤16	>16
Normal	15	4	6	2
Abnormal	41	3	13	5
Total(Data av.^)	56	7	19	7
**123 missing (63.1%); ^105 missing (64.0%); *blood, urine or cerebrospinal fluid; av., available*				

Supplemental Table 2. Laboratory findings by age at onset

**Treatment by age at onset**				
**Treatment**	**Age at onset**			
	0-≤3 (39 pts)	>3-≤6 (3 pts)	>6-≤16 (15 pts)	>16 (13 pts)
CBZ	3	1	1	1
LTG	0	0	5	2
VPA	22	2	13	1
OXC	2	0	6	0
TPM	3	0	4	1
CLB	2	0	0	2
CZP	3	0	5	2
GVG	0	0	0	0
PHT	6	1	5	5
PB	7	1	4	2
DZP	4	0	3	1
LZP	5	1	0	0
LEV	8	0	3	4
LCM	0	1	0	0
ZNS	0	0	1	0
GBP	0	0	1	1
Steroids	3	0	1	0
Midazolam	5	1	4	3
Tiopentale	5	0	3	1
Propofol	2	0	0	2
Ketogenic diet	2	1	0	2
Ig-iv	2	0	0	0
Pyridoxine	1	0	0	0
Riboflavin	1	0	0	0
Carnitine	1	0	2	0
Q10	1	0	1	0
Magnesium	0	0	0	2
Paraldehyde	1	0	0	0

CBZ, carbamazepine; LTG, lamotrigine; VPA, valproic acid; OXC, oxcarbazepine; TPM, topiramate; CLB, clobazam CZP, clonazepam; GVG, vigabatrine; PHT, phenytoin; PB, phenobarbital; DZP, diazepam; LZP, lorazepam LEV, levetiracetam; LCM, lacosamide; ZNS, zonisamide; GBP, gabapentin; Ig-iv, intravenous Immunoglobulins

Supplemental Table 3. Treatment by age at onset

Survival by age at onset					
**Survival**	**Total**	**Age at onset**			
		0-≤3	>3-≤6	>6-≤16	>16
≤1y	57	42	3	7	5
> 1y - ≤5 y	16	10	0	6	0
> 5y - ≤ 10 y	16	6	1	7	2
> 10y - ≤15 y	12	5	0	5	2
> 15 y	15	0	3	6	6
Alive at f-u	55	13	3	21	18
Total (Data available*)	171/195	76	10	52	33
** 24 missing (12.3%); y, years; f-u, follow-up*					

Supplemental Table 4. Survival by age at onset
